# Altered Nuclear Expression of the Deubiquitylase BAP1 Cannot be Used as a Prognostic Marker for Canine Melanoma

**DOI:** 10.1016/j.jcpa.2018.06.007

**Published:** 2018-07

**Authors:** N. Jama, N. Farquhar, Z. Butt, S.E. Coupland, J.J. Sacco, T. Scase, A.B. Fielding, J.M. Coulson, H. Kalirai, D.R. Killick

**Affiliations:** ∗ Department of Small Animal Clinical Sciences, Institute of Veterinary Science, University of Liverpool, Liverpool, UK; † Molecular and Clinical Cancer Medicine, Institute of Translational Medicine, University of Liverpool, Liverpool, UK; ‡ Cellular and Molecular Physiology, Institute of Translational Medicine, University of Liverpool, Liverpool, UK; § Bridge Pathology Ltd., 637 Gloucester Road, Bristol, UK

**Keywords:** BAP1, dog, immunohistochemistry, melanoma

## Abstract

BRCA1-associated protein-1 (BAP1) is a nuclear localized deubiquitylating enzyme that belongs to the ubiquitin c-terminal hydrolase subfamily. The encoded protein is highly homologous between man and dogs, suggesting a functional significance preserved by evolution. BAP1 has multiple properties, including tumour suppressor activity. Loss of BAP1 function is implicated in the oncogenesis of several types of cancers including uveal, mucosal and some cutaneous melanomas in humans, as well as in mesothelioma. In this study we investigate the significance of BAP1 in canine melanoma. Nuclear BAP1 protein was detected in five canine oral melanoma cell lines using an antibody commonly used for analysis of human tissues. BAP1 loss of function mutations often lead to loss of nuclear BAP1 (nBAP1) expression in humans; this is associated with a poorer prognosis in uveal and mucosal melanoma. Therefore, as a prelude to a study evaluating the prognostic significance of nBAP1 expression in dogs, immunohistochemistry (IHC) was used to assess cases of canine melanoma for nBAP1 expression. In 89 cases where tumour cells were identified by melan-A labelling, 100% of tumour cells were positive for nBAP1 expression, including eight uveal tract and 29 oral mucosal melanomas. This finding indicates that BAP1 IHC cannot be used as a prognostic marker in canine uveal and mucosal melanoma. Moreover, this observation suggests that either BAP1 has a different functional significance in canine melanoma or that loss of BAP1 function is achieved by a different route. This is a novel finding that warrants further investigation to determine the comparative biological relevance.

## Introduction

Ubiquitin (Ub) is an 8.5 kDa (kilodalton) protein that ‘addresses’ proteins for degradation or trafficking to a particular cellular site; additionally, it is involved in regulating essential transcriptional factors such as p53 (Hershko et al., 2000, Hochstrasser, 2000, Pickart, 2001, Komander et al., 2009, Sun and Dai, 2014). Ubiquitylation involves a catalytic cascade by E1 (ubiquitin activating), E2 (ubiquitin conjugating) and E3 (ubiquitin ligase) enzymes to form an isopeptide bond between a lysine residue on the target protein and Ub (Hochstrasser, 2000). Modification can result in either mono-ubiquitylation on the addition of a single Ub moiety or poly-ubiquitylation on the addition of Ub chains (Hochstrasser, 2000, Pickart, 2001, Komander, 2009, Komander et al., 2009). An important aspect of ubiquitylation is the ability to reverse the process by removing Ub moieties from the substrate through the action of deubiquitylating enzymes (DUBs) (Nijman et al., 2005, Komander et al., 2009, Clague et al., 2012, Abdul Rehman et al., 2016). Depending on their catalytic domains, DUBs are divided into six families (Nijman et al., 2005, Komander et al., 2009, Clague et al., 2012, Abdul Rehman et al., 2016). Five belong to the cysteine protease family and include ubiquitin-specific proteases (USPs), ubiquitin c-terminal hydrolases (UCHs), ovarian tumour proteases (OTPs), Josephins and MINDYs (Nijman et al., 2005, Komander et al., 2009, Clague et al., 2012, Abdul Rehman et al., 2016). The sixth family are zinc metalloproteases known as JAB1/MPN/MOV34 (JAMMs) (Nijman et al., 2005, Komander et al., 2009, Clague et al., 2012, Abdul Rehman et al., 2016).

**Fig. 1:**
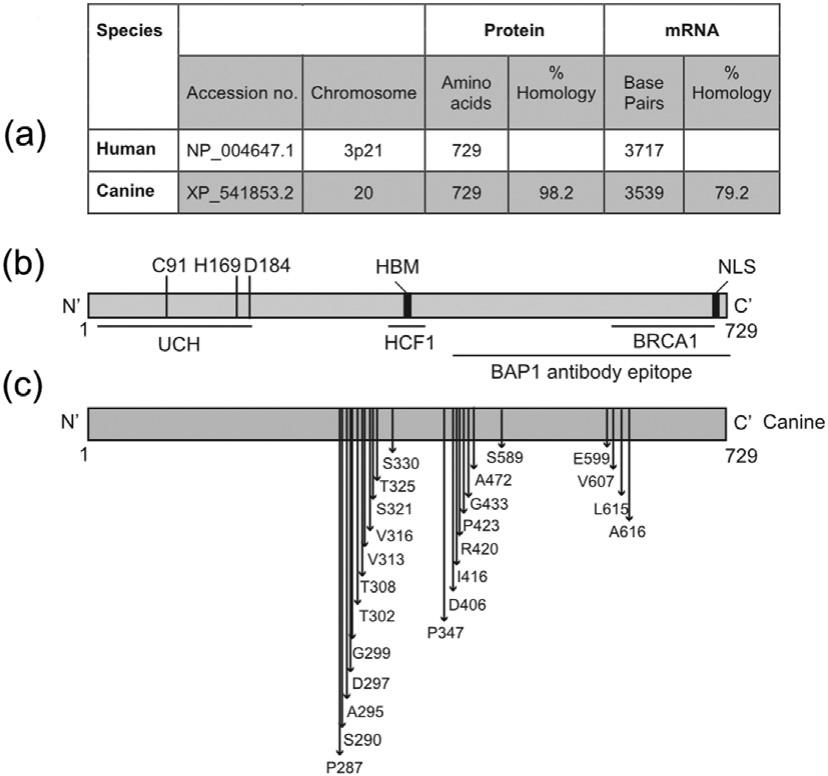
(a) Comparative sequence information for canine and human *BAP1* genes, mRNAs and proteins, showing that canine and human BAP1 are highly homologous. (b) Schematic diagram of human BAP1 protein (NP_004647.1) featuring key functional domains and protein interaction sites: ubiquitin C-terminal hydrolase (UCH) domain highlighting the catalytic triad (C91, H169, D184), host cell factor-1 (HCF1) binding motif (HBM) and a nuclear localization signal (NLS). (c) The canine BAP1 protein (XP_541853.2) was aligned to the human sequence; amino acid polymorphisms between human and canine BAP1 are highlighted by arrows.

BRCA1-associated protein-1 (BAP1) is a DUB that belongs to the UCH subfamily (Jensen *et al.*, 1998). In humans, the *BAP1* gene is located on chromosome 3p21.3. It encodes a protein consisting of 729 amino acids (Fig. 1a). BAP1 consists of an N-terminal UCH domain containing a catalytic triad, a host cell factor-1 (HCF1) binding motif (HBM) and a nuclear localization signal (NLS) at the C-terminus (Fig. 1b) (Jensen *et al.*, 1998). Initially BAP1 was identified in a yeast two-hybrid screen as a BRCA1-binding protein due to its interaction with the RING finger domain of this tumour suppressor (Jensen *et al.*, 1998), and was shown to have an inhibitory effect on the growth of MCF7 breast cancer cells, indicating that BAP1 acts as a tumour suppressor gene (Jensen *et al.*, 1998).

BAP1 is a nuclear localized DUB that interacts with other proteins, often as part of a multiprotein complex including HCF1 and ASXL1/2, which is involved in regulating essential cellular processes including transcription (Jensen et al., 1998, Dey et al., 2012). BAP1 can remove multiple ubiquitin molecules from substrates such as HCF1 to stabilize them, and can remove single ubiquitin molecules from histone H2A (Machida et al., 2009, Misaghi et al., 2009, Sacco et al., 2015). BAP1 has been shown to undergo both somatic and germline mutation, the latter underpinning a familial cancer predisposition syndrome (Abdel-Rahman et al., 2011, Testa et al., 2011). *BAP1* mutations commonly lead to loss of its nuclear expression, as nonsense or frameshift mutations lead to truncated BAP1 protein lacking the NLS, and/or reduced protein or mRNA stability. Such loss of nuclear BAP1 is seen in a variety of human cancers, most commonly malignant mesothelioma, uveal melanoma, renal clear cell carcinoma and some cutaneous melanomas (Harbour et al., 2010, Abdel-Rahman et al., 2011, Carbone et al., 2012, Song et al., 2017). In humans, loss of nuclear BAP1 (nBAP1) is common in oral mucosal and uveal melanoma, but uncommon in cutaneous melanoma (Kalirai et al., 2014, Song et al., 2017). In uveal melanoma, mutation of the *BAP1* gene is associated with loss of nBAP1 expression, which has been proposed as a surrogate clinical marker (Kalirai et al., 2014, Koopmans et al., 2014). Loss of nBAP1 (assessed by immunohistochemistry [IHC]) is clinically significant in human oral mucosal and uveal melanoma, where it is noted in around 45% and 50% of cases, respectively, and has been linked to a poor prognosis (Kalirai et al., 2014, Song et al., 2017).

Melanoma is an aggressive tumour that affects both humans and domestic dogs. In both species, melanoma may arise at a number of different anatomical sites (Smith et al., 2002, Gillard et al., 2014, Schiffman and Breen, 2015). Melanoma in dogs develops in similar anatomical regions to the tumour in man, albeit with different frequency at each site. For example, cutaneous melanoma, the most common location in man, is much less common in dogs, with forms analogous to human mucosal and acral lentiginous melanoma being relatively more common (Smith et al., 2002, Gillard et al., 2014, Schiffman and Breen, 2015).

The aim of this study was to examine BAP1 involvement in canine cancers. We set out to characterize canine BAP1 and to compare nBAP1 protein expression in canine melanoma with that previously described in human melanoma. Specifically, we hypothesized that loss of nBAP1 in canine oral and uveal melanoma would be associated with a poorer prognosis. As a prelude to the prognostic study we evaluated nBAP1 expression in a tissue microarray (TMA) composed of canine melanoma from a variety of sites.

## Materials and Methods

### Alignment Analysis

To determine the degree of homology between canine (gene ID 484737) and human (gene ID 8314) *BAP1* mRNA and protein, sequences were acquired from the National Center for Biotechnology Information (www.ncbi.nlm.nih.gov) and aligned using CLC Main workbench system (version 7.6.4, Qiagen, Denmark).

### Cell Culture

Human mesothelioma cell lines MSTO-211H and NCI-H28 were cultured as described by Sacco *et al.* (2015). Canine malignant melanoma cell lines from five dogs were a generous gift from Dr. M. S. Kent at the University of California Davis, Davis, California, USA. UCDK9M3 and UCDK9M4 are from primary oral mucosal melanomas, UCDK9M2 and UCDK9M5 are from lymph node metastases of oral mucosal melanoma and UCDK9M1 was derived from a skin metastasis of an oral mucosal melanoma (Aina *et al.*, 2011). These cell lines were cultured in Dulbecco's modified Eagle's medium (DMEM) with 10% heat inactivated fetal bovine serum and 1% non-essential amino acids. Cells were confirmed as mycoplasma free and were passaged regularly by washing with phosphate buffered saline, detaching with trypsin for 2–3 min at 37°C and reseeding in fresh media. All cells were grown in a humidified incubator at 37°C and 5% CO_2_.

### RNA Interference

UCDK9M4 cells were seeded at a density of 1.2 × 10^5^/well into a six-well plate. The following day, they were transfected with 40 nM siRNA: hs_BAP1_2, hs_BAP1_5 (Qiagen) or a non-targeting siRNA control (siC) using Oligofectamine (Invitrogen, Carlsbad, California, USA) following the manufacturer's protocol. After 72 h, transfected cells were harvested for protein extraction and evaluation by immunoblotting. The siRNAs were designed to target human BAP1; however, hs_BAP1_2 and hs_BAP1_5, have 100% and 79% homology with the canine sequence, respectively.

### Immunoblotting

Whole cell protein extracts were prepared by scraping cells directly into hot Laemmli buffer (50 mM Tris pH 6.8, 2% sodium dodecyl sulphate [SDS], 10% glycerol) and incubated at 110°C for 10 min with vortexing every 2 min. The protein concentration of each sample was established using a bicinchoninic acid assay (Thermo Scientific, Rockford, Illinois, USA). Protein extracts were prepared in Laemmli buffer and denatured at 98°C for 5 min before electrophoresing on 10% SDS polyacrylamide gel electrophoresis gels and subsequent transfer onto BiotraceNT membrane (VWR International Ltd., Lutterworth, UK). Membranes then underwent blocking, washing and primary antibody incubation overnight at 4°C, followed by washing (in Tris-buffered saline with 0.1% Tween-20) and secondary antibody incubation for 1 h at room temperature. Primary antibodies used were mouse anti-human BAP1 (C-4, Santa Cruz Biotechnology, Santa Cruz, Californian, USA) and mouse anti-human β-actin (Abcam, Cambridge, UK). To visualize proteins, donkey anti-mouse IRDye secondary antibodies were used for detection (LI-COR Biosciences UK Ltd., Cambridge, UK) using a LI-COR Odyssey Classic blot imager and images were evaluated using Image Studio™ (LI-COR).

### Immunofluorescence

Canine melanoma cells (UCDK9M1, UCDK9M3 and UCDK9M5) were seeded onto autoclaved coverslips in six-well plates at 1.5 × 10^5^/well. The following day, medium was aspirated and the cells were fixed in 4% paraformaldehyde, quenched with ammonium chloride and permeabilized with 0.1% triton prior to blocking and incubation with the mouse anti-human BAP1 primary antibody (Santa Cruz). Alexa-Fluor 488-conjugated secondary anti-mouse antibody (Molecular Probes, Eugene, Oregon, USA) was used. Coverslips were mounted on Moviol (Sigma-Aldrich, St. Louis, Missouri, USA) supplemented with 4′,6-diamidino-2-phenylindole (DAPI; Sigma-Aldrich) at 1 in 10,000 dilution where indicated. Cells were imaged using a Nikon Eclipse Ti (CFI Plan Apochromat 40× NA 0.95, WD 0.14 mm or CFI Super Plan Fluor ELWD ADM 20×C NA 0.45, WD 8.2–6.9) microscope (Nikon, Tokyo, Japan) and data analysed using Fiji software (Schindelin *et al.*, 2012).

### Clinical Case Selection

Canine melanoma tissue samples were sourced from clinical cases submitted to Bridge Pathology. In each case, a diagnosis of melanoma was made by a board-certified veterinary pathologist. Cases were reviewed to determine whether they were suitable for IHC.

### Construction of a Tissue Micro-array and Immunohistochemistry

A tissue micro-array (TMA) containing triplicate 0.6 mm tissue cores from each case was constructed using the manual tissue micro-arrayer (Beecher Instruments, Sun Prairie, Wisconsin, USA) as described by Kalirai *et al.* (2014). Samples included in the TMA were grouped as follows: digit, skin, oral, uveal and periocular. Sections (4 μm) were cut from the TMA block and mounted on slides.

The TMA sections underwent antigen retrieval using the Dako pretreatment module (Agilent Technologies LDA UK Ltd., Stockport, UK); slides were incubated in a high-pH bath containing EnVision™ FLEX target retrieval solution (Tris/EDTA buffer pH 9.0) at 96°C for 20 min. IHC was performed on the Dako Autostainer PLUS machine, using the Dako Envision™ FLEX Kit (Agilent) according to the manufacturer's instructions. Slides were incubated with the following antibodies for 30 min: mouse anti-human melan-A (clone A103, Agilent) at a concentration of 1 μg/ml to aid identification of melanoma cells; and mouse anti-human BAP1 at a concentration of 1 μg/ml. Positive labelling was ‘visualized’ with 3,3′ diaminobenzidine for BAP1 and amino-ethyl carbazole (Vector Laboratories, Peterborough, UK) for melan-A. The sections were counterstained with haematoxylin. Human pancreas was used as a positive control to demonstrate nuclear BAP1 (nBAP1) localization and human ocular melanoma tissue was used as a positive control for melan-A. Additional sections were treated with mouse IgG1 isotype control at the same concentration as the BAP1 and melan-A primary antibodies and served as negative controls.

### Scanning and Scoring

The TMA slides were scanned using an Aperio Imagescope (Leica Biosystems, Wetzlar, Germany) and assessed for nBAP1 protein expression by three independent observers (NJ, HK and SC). The tissue samples were given a final score according to the percentage of tumour nuclei that expressed nBAP1 protein across the TMA cores (Kalirai *et al.*, 2014).

### Ethical Approval

Ethical approval for this project was granted by the University of Liverpool Committee to the Faculty of Health and Life Sciences on Research Ethics (Veterinary Science) – project number VREC360.

## Results

### Canine and Human BAP1 are Highly Homologous

Initially, alignments of protein (canine XP_541853.2, human NP_004647.1) and mRNA (canine XM_541853.5, human NM_004656.3) were generated (Fig. 1a) and the homology between human and canine BAP1 was evaluated (Fig. 1b). The human mRNA and predicted canine mRNA were highly similar, and the predicted canine BAP1 protein was virtually identical to that of the human BAP1 at 98% amino acid homology. Alignment analysis showed that those amino acids differing between human and canine BAP1 clustered in three areas; notably these were all outside the major functional domains, which were conserved (Figs. 1b and c).

### BAP1 Antibody Validation and BAP1 Expression in Canine Melanoma Cell Lines

**Fig. 2:**
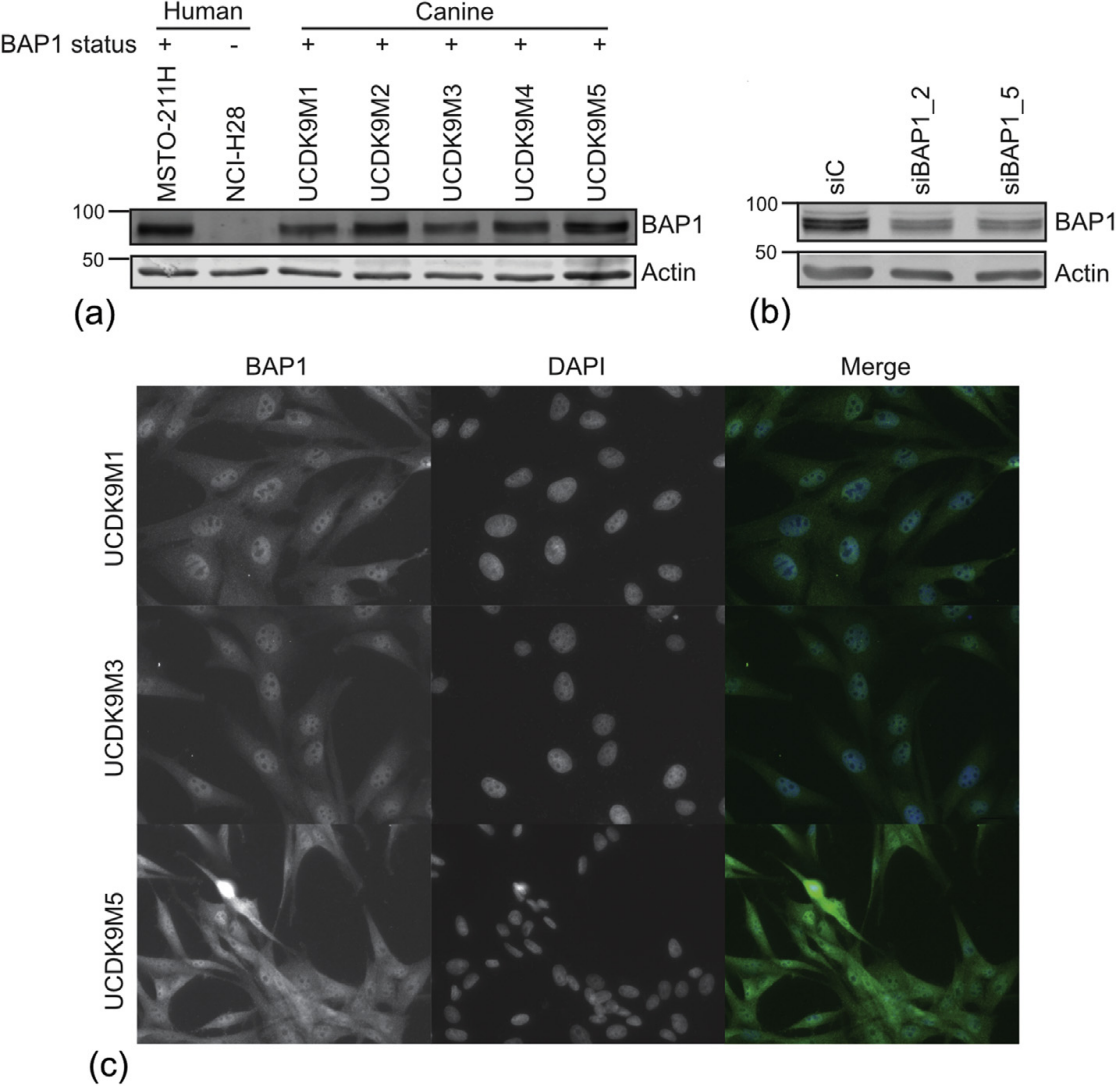
(a) BAP1 expression was assessed by immunoblotting using whole cell extracts from five canine melanoma cell lines and the human mesothelioma cell lines MSTO-211H (BAP1 positive) and NCI-H28 (BAP1 negative); β-actin was used as a loading control. (b) Knockdown using two BAP1 targeting siRNAs leads to reduced expression of BAP1 protein in UCDK9M5 canine melanoma cells. (c) Three canine oral melanoma cell lines (UCDK9M1, UCDK9M3 and UCDK9M5) were immunolabelled using the same human anti-BAP1 antibody and counterstained with DAPI; canine BAP1 demonstrated a predominantly nuclear localization. Bar, 120 μm.

In this study we used a BAP1 antibody, which is commonly reported in the literature as detecting BAP1 in human cell lines and human tissue samples (Kalirai et al., 2014, Sacco et al., 2015). The epitope for this antibody is largely conserved between human and canine BAP1 sequences, suggesting likely cross reactivity (Figs. 1b and c). To confirm this, and to examine BAP1 expression in the canine melanoma cell lines, immunoblotting was performed (Fig. 2a). The human mesothelioma cell lines MSTO-211H (BAP1 positive) and NCI-H28 (BAP1 negative) (Sacco *et al.*, 2015) were used as controls for BAP1 immunoreactivity, and β-actin was used as a loading control. A strongly immunoreactive band of around 85 kDa, the same molecular weight as human BAP1, was detected in each of the five canine oral melanoma cell lines examined.

To confirm the identity of this immunoreactive band, we ‘knocked down’ BAP1 in the UCDK9M5 cell line, using two BAP1 siRNA sequences independently (Fig. 2b). These siRNAs are designed against the human sequence and targeted regions with high sequence homology between the two species (siRNA HS_BAP1_2 is an exact match); both siRNAs resulted in 80% reduction in the BAP1 immunoreactive band compared with a non-targeting siRNA. In order to confirm that BAP1 localized to the nuclei of canine melanoma cell lines, we used immunofluorescence (Fig. 2c). In the three cell lines examined, BAP1 showed a predominantly nuclear localization, with exclusion from the nucleoli and some diffuse cytoplasmic labelling. Together these data suggest that oral canine melanoma cell lines express nBAP1 protein, and that this anti-human BAP1 antibody can be used for IHC in canine melanoma tissue.

### Histological Assessment of the Tissue Microarray Sections

One-hundred and five samples were retrieved initially; however, following HE staining, 15 samples were excluded due to inadequate preservation or excessive pigmentation that would have interfered with IHC. Therefore, 90 samples (Table 1) were arrayed on the TMA.

**Table 1:** Distribution of melanoma sites, age and gender

Anatomical site (number of cases)	Median age (Range)	Gender	
		Male	Female
Digit (19)	9y 7m (6y 2m–13y)	16	3
Skin (22)	8y 7m (1y 6m–13y 3m)	12	10
Oral (28)	11y 8m (8y–16y)	17	11
Uveal (8)	10y 3m (5y–13y)	3	5
Periocular (13)	9y 5m (6y–13y)	6	7

### Clinical Data

**Fig. 3:**
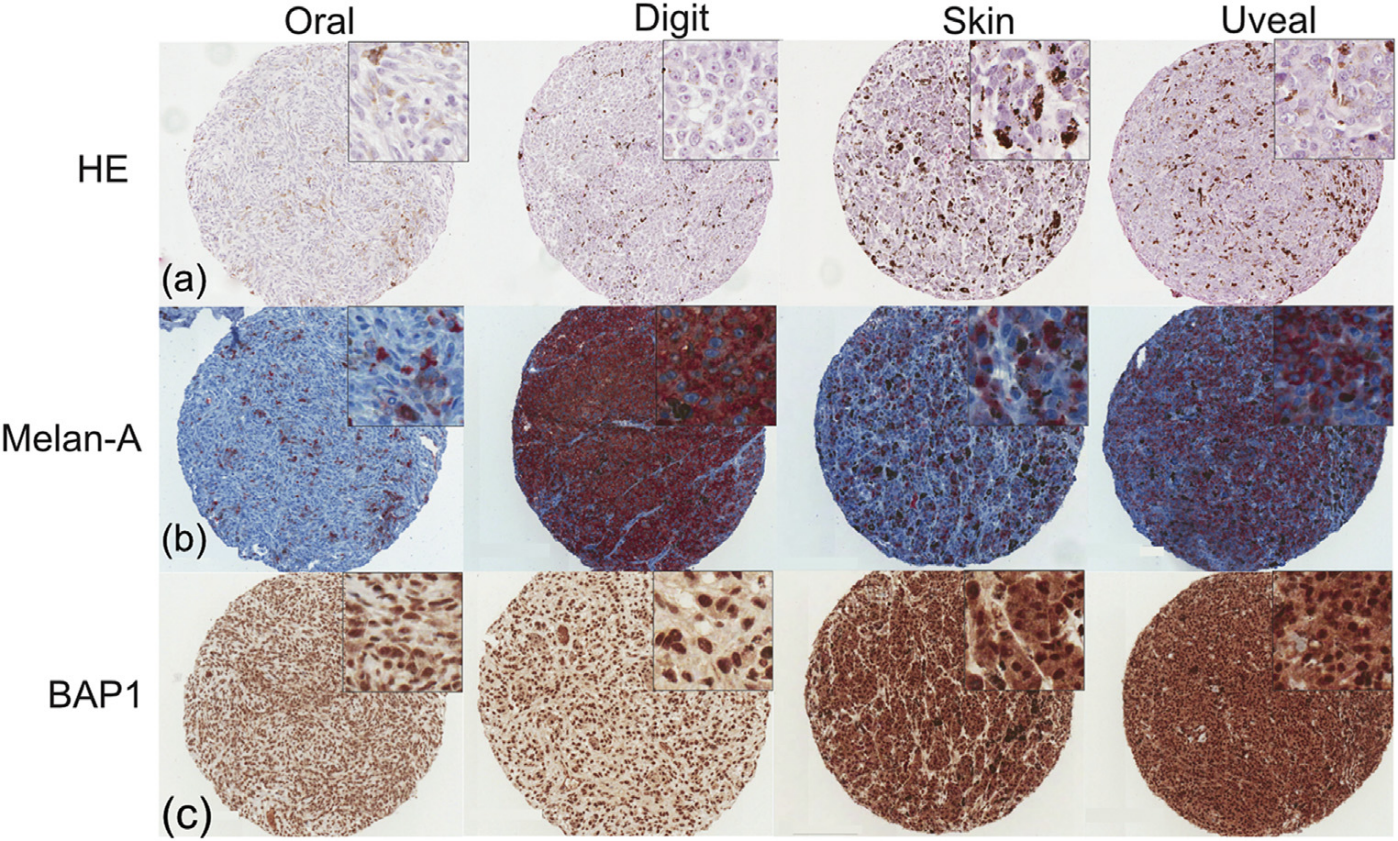
Representative images are shown for four cores from the following anatomical sites: oral, digit, skin and uveal. Main images are at ×8 magnification, insets are at ×40. (a) HE-stained TMA sections used to determine tumour regions. (b) Melan-A labelled TMA sections used to identify melanocytic tumour cells. (c) BAP1 immunoreactivity in TMA sections.

The clinical characteristics of the 90 canine samples included in the study are summarized in Table 1. They comprised of 37 females and 53 males. The cases came from animals of 25 different breeds and included 17 crossbred dogs; breeds represented by more than five individuals included: cocker spaniel (*n* = 8), German shepherd dog (*n* = 6), golden retriever (*n* = 7), Labrador retriever (*n* = 15), Rottweiler (*n* = 7). The median age at diagnosis was 9 years. These dogs were diagnosed with melanoma, which originated from different anatomical sites including: digits (*n* = 19), skin (*n* = 22), oral cavity (*n* = 28), uveal tract (*n* = 8) and periocular tissue (*n* = 13; seven eyelid and six conjunctival) based on histopathological examination by a board-certified veterinary pathologist (TS) (Fig. 3a).

### Assessment of BAP1 Expression by Immunohistochemistry

Melan-A labelling was performed in order to confirm the diagnosis of melanoma as well as to identify the location of the melanoma cells in the tissue. In some cases, melanoma cells made up the majority of cells in the tissue core (Fig. 3b, digital and uveal). In other cases, there were islands or sporadic melanoma cells throughout the core (Fig. 3b, skin and oral). On initial labelling, three cases were only focally faintly positive for melan-A and four were melan-A negative. The seven cases with either missing tumour or little/no melan-A labelling, underwent sectioning of the whole tissue block and were re-labelled in the study. Of these seven cases, six labelled positively for melan-A, while one case remained negative for melan-A expression. All cases were included in the subsequent analysis.

Of the 90 canine samples with confirmed melanoma, 89 showed positive nBAP1 labelling (Fig. 3c, Table 2). However, one case labelled only very faintly focally positive for nBAP1 and interestingly this was the same case that labelled negatively for melan-A. In addition, strong to weak cytoplasmic BAP1 (cBAP1) expression was also seen in each case, consistent with the immunofluorescence data for canine melanoma cell lines (Fig. 2c). Overall, with the exception of the single melan-A negative case, strong nBAP1 labelling was seen in each of the samples drawn from the most common anatomical sites to be affected by melanoma in the dog (Fig. 3c).

**Table 2:** Frequency of nuclear BAP1 labelling

Anatomical site	Melan A-positive tumour cells	100% of tumour cells nBAP1 positive
Digit	18/19 + 1 equivocal	18/19
Skin	22/22	22/22
Oral	28/28	28/28
Uveal	8/8	8/8
Periocular	13/13	13/13

## Discussion

Loss of function of the tumour suppressor gene BAP1 is believed to play a role in the progression of several human cancers including uveal and oral mucosal melanoma and some cutaneous melanomas (Jensen et al., 1998, Andrici et al., 2016). Dogs are also affected by each of these melanoma types (Gillard *et al.*, 2014). Here we highlight the marked homology between human and canine BAP1, showing that complete sequence identity is conserved within the major functional domains. Although alignments reveal several amino acid polymorphisms, these are tightly clustered outside the functional domains, and at present the impact, if any, that these may have on the function of the canine BAP1 protein is unknown. Detailed mechanistic analysis will be needed to assess whether these sequence differences confer any differential BAP1 functionality across species.

In this study, 90 canine melanomas obtained from the digits, skin, oral cavity, uveal tract or the periocular region were analysed for nBAP1 expression using IHC. Lack of nBAP1 protein expression is associated with loss of function mutations of BAP1 and is common in several human cancers such as uveal and oral mucosal melanoma and malignant mesothelioma. Functional studies have demonstrated that loss of IHC expression for nBAP1 is a useful marker of clinical behaviour in these tumours (Kalirai et al., 2014, Andrici et al., 2016, Song et al., 2017). In this study of 90 melanoma cases, the 89 cases that were melan-A positive labelled with 100% positivity for nBAP1 protein expression within the tumour cells. We have not ascertained why the remaining case from a digital melanoma had only equivocal nBAP1 expression; however, this case also labelled negatively for melan A. This likely indicates a technical issue as this sample underwent decalcification, which is known to reduce antibody binding (Ramos-Vara and Miller, 2011).

This study was performed in the same laboratory using the same technique as a previous study that reported that loss of nBAP1 is associated with a poorer outcome in human uveal melanoma (Kalirai *et al.*, 2014). Moreover, the work presented here was performed contemporaneously with, and with the same methodology as, a follow-up study (to Kalirai *et al.*, 2014) in this laboratory, which confirmed the prognostic significance of loss of nBAP-1 in human uveal melanoma (Farquhar *et al.*, 2018). The possibility that the unexpected results of this study relate to experimental methodology can therefore be excluded.

The results of this study indicate that the incidence of nBAP1 protein loss in canine melanoma differs from that reported in human melanoma, specifically uveal and oral mucosal melanoma where loss of nBAP1 protein expression is reported in around 50% of cases (Kalirai et al., 2014, Song et al., 2017). This loss is correlated strongly with a poor prognosis (Kalirai et al., 2014, Song et al., 2017). Furthermore, the absence of nBAP1 protein expression is indicative of the presence of a *BAP1* mutation in human cancers such as malignant mesothelioma and uveal melanoma, as well as Spitz melanoma (Wiesner et al., 2012, Yoshikawa et al., 2012, Song et al., 2017). A more recent study has shown that both nBAP1 and cBAP1 expression were absent in 70% of uveal melanoma patients who developed metastasis (Kalirai *et al.*, 2014). As a result, several studies have proposed routine inclusion of IHC for BAP1 when investigating uveal melanoma and malignant mesothelioma (Koopmans et al., 2014, Cigognetti et al., 2015, Andrici et al., 2016).

Almost nothing is known about the biology of BAP1 (and DUBs more generally) in dogs, and while one possibility is that BAP1 is not of significance in canine melanoma, another is that canine melanoma reaches the same functional outcome by a different route not detectable by our approach. For example, it has been shown that retention of nBAP1 protein expression does not necessarily exclude the possibility that the *BAP1* gene may harbour loss of function mutations (Ventii *et al.*, 2008). Ventii *et al.* (2008) demonstrated that missense mutations within the catalytic domain of BAP1 reduced tumour suppressor activity, but not protein expression, in a human NSCLC cell line. This highlights the fact that the presence of *nBAP1* protein expression does not rule out the possibility of abnormalities in *BAP1* function *per se*. Of note, although *BAP1* knockout mice develop a myelodysplastic disorder with features of chronic myelomonocytic leukaemia (CMML), in human CMML, mutation of *BAP1* is rare but that of the BAP1 interacting protein ASXL1 is very common (Dey *et al.*, 2012).

In conclusion, the present study has validated reagents for investigation of BAP1 in canine cancer. Using these reagents we undertook the first evaluation of BAP1 in dogs. We investigated nBAP1 expression by IHC in a panel of canine melanoma samples from different anatomical sites; this was planned as a prelude to a study investigating the prognostic significance of BAP1 in canine melanoma. Unexpectedly, all of the samples expressed nBAP1, therefore we conclude that BAP1 IHC cannot be used for prognostication in canine melanoma. This is a novel finding and warrants further investigation as part of the ongoing effort to identify new biomarkers and treatments for canine melanoma and development of canine melanoma as a model for the disease in man.
